# Thriving after cancer: a special collection on the benefits of exercise and nutrition in cancer survivorship

**DOI:** 10.1093/jncics/pkaf048

**Published:** 2025-06-11

**Authors:** Leila Tchelebi, Justin Brown

**Affiliations:** Northwell, New Hyde Park, NY, United States; Director, Exercise and Cancer Biology Research Program, AdventHealth, Orlando, FL, United States

There exists a body of research demonstrating that a healthy diet and exercise positively affect cancer outcomes.^1-4^ Nutrition and exercise have been linked to improved outcomes in a variety of cancer domains including mitigating treatment side effects, improving quality of life, and reducing both cancer and noncancer mortality.^2^^,^^5^ However, the existing literature is often observational or preclinical, which may create uncertainty regarding the causality of the conclusions or how such findings affect real-world cancer patients. Further, there remain several unanswered questions as to how to engage patients in these health-promoting behaviors, which patients benefit and whether these benefits persist in the real world, what the mechanisms are behind these improved outcomes, and how to objectively measure healthy eating and exercise. *JNCI Cancer Spectrum* is thus calling for papers investigating how exercise and nutrition affect cancer outcomes so that we may begin to fill these gaps in knowledge.

Knowledge that a treatment is effective is only part of the battle when it comes to cancer care. Equally important is convincing patients to undergo the recommended therapy. This is particularly true when the treatment must be self-administered by the patient, as with diet and exercise, rather than delivered by a provider via a linear accelerator or infusion pump. The onus thus falls on the provider to better understand how to motivate patients to maintain proper nutrition and to exercise after a cancer diagnosis. As providers, we must develop an understanding of the psychology behind the behavior change to successfully motivate patients to engage in these therapeutic interventions.^6^ Research is needed on how best to communicate the benefits of a healthy diet and exercise to patients such that they will be more apt to engage in these behaviors.

Existing data on exercise has focused on cancers with a relatively good prognosis, in relatively healthy patients.^7-10^ Would the same benefits persist in other cancer patients, such as those with pancreatic cancer, soon to be the third leading cause of cancer death, in which any intervention that may improve mortality should be encouraged?^11^ Is exercise feasible in these patients who suffer significant weight loss by the time of diagnosis and tend to develop severe cachexia and malnutrition? ^12^ Do the therapeutic benefits of nutrition and exercise pertain only to certain patients with particular clinical and pathological characteristics, as has been seen in breast cancer? ^13^

Once recommended, how can we ensure that patients are complying with the recommended interventions? To demonstrate that diet and exercise are the causal agents in improving patient outcomes, it is imperative that we ascertain whether patients complied with the recommended interventions. We must also understand the mechanisms by which diet and exercise improve cancer outcomes. Do diet and exercise affect cellular proliferation, gene expression, biomarkers, or oxygenation of cells? Do the effects of diet and exercise on cells apply only to specific therapies, such as radiation, which is known to work best in oxygenated cells?^14^ Research in this area is underway, but there is still much to discover.^10^^,^^15^^,^^16^

The cost of cancer care has grown exponentially over the last several decades and is estimated to increase 34% by 2030.^17^ Meanwhile, Phase 1 clinical trial data has demonstrated that walking approximately 225 minutes per week is safe and feasible for prostate cancer patients,^9^ and can be done while incurring minimal (if any) health-care costs. In an era of economic uncertainty such as the present, it is critical to emphasize therapies that are cost-effective for our cancer patients. However, are improved nutrition and exercise cost-effective? If exercise is recommended to a patient population that not only does not benefit but that may be harmed by the intervention, could these interventions ultimately be adding to the costs of our health-care system?

In summary, our understanding of how nutrition and exercise affect cancer outcomes has only reached the tip of the iceberg. The Editors of *JNCI Cancer Spectrum* thus encourage literature aimed at gaining a more specific and granular understanding of how these interventions benefit patients, which patients benefit most, how we can measure that the interventions were upheld, and whether these interventions can be applied to patients in the real world. We particularly encourage studies that involve clinical trials or prospective data collection over papers that are exploratory and analyze existing data for statistical associations without a well-considered biological mechanism.

## Data Availability

Not applicable.
